# ER-resident proteins are key players in cartilage and bone homeostasis

**DOI:** 10.3389/fcell.2025.1661846

**Published:** 2025-11-07

**Authors:** Sina Stücker, Yvonne Rellmann, Sandra Schulte, Rita Dreier

**Affiliations:** Institut of Physiological Chemistry and Pathobiochemistry, University of Münster, Münster, Germany

**Keywords:** cartilage, extracellular matrix, endoplasmic reticulum, chaperone, protein folding, ER stress

## Abstract

Hyaline cartilage is essential for bone formation and joint function. It contains a dense extracellular matrix that is produced in the ER of chondrocytes. Therefore, the ER contains a complex machinery of enzymes including chaperones, glycosyltransferases and hydroxylases that control folding, modification and secretion of newly synthesized matrix proteins. Loss or malfunction of ER-resident chaperones and proteins leads to misfolding and accumulation of matrix proteins in the ER. This causes ER stress and disrupts crucial cellular processes including chondrocyte differentiation, signaling and matrix production. During skeletal development, deficiency of ER chaperones disrupts cartilage and bone formation by impairing the folding and maturation of collagens and other matrix proteins, causing chondrodysplasia, pseudoachondroplasia and other skeletal diseases. Loss of ER-resident chaperones also impairs the integrity and stability of the cartilage matrix, promoting its degeneration during osteoarthritis. Due to the complexity of the ER protein processing machinery, the specific roles of ER-resident proteins in cartilage and bone homeostasis largely remain elusive. This review provides an overview of the most common ER-resident proteins and our current understanding of their function in cartilage homeostasis and disease.

## 1 Introduction

### 1.1 Cartilage homeostasis

Cartilage is a specialized matrix-rich tissue of the skeletal system. In the mammalian body, there are three different types of cartilage, including elastic cartilage, fibrocartilage and hyaline cartilage. Each of these types has a distinct location, structure and function. Elastic cartilage can be found in the ear lobes, larynx and trachea, providing shape and elasticity (Nasiri et al., 2023). Fibrocartilage is the stiffest type of cartilage. It is present in the meniscus, symphysis and at the transition from tendon to bone, providing stability and shock absorbance (Fox et al., 2012). Hyaline cartilage is the most abundant type of cartilage. It is mainly located in joints, where it is also referred to as articular cartilage. Here, it covers the bone ends to provide a smooth surface for joint movement (Krishnan and Grodzinsky, 2018). During skeletal development, hyaline cartilage can also be found in the bone anlagen and in the growth plate where it serves as a temporary scaffold for bone formation (Melrose et al., 2016). Eventually, the cartilaginous portion of the growth plate is replaced by bone. Hyaline cartilage is composed of a dense extracellular matrix (ECM) that contains crosslinked fibrils of collagens type II, IX and XI. This fibrillar network embeds additional collagens and proteoglycans such as aggrecan and other glycoproteins (Mendler et al., 1989). Due to their negative charge, proteoglycans are strongly hydrophilic, binding water within the fibrillar collagen network. This provides lubrication, tensile strength and resistance to compressive loads and gives the cartilage its unique viscoelastic structure and biomechanical properties of articular cartilage (Sophia Fox et al., 2009). Structurally, hyaline cartilage in growth plates and on bone surfaces within joints can be divided into different layers, which directly relate to its function. This zonal organization is mainly based on matrix composition and the phenotype of the cartilage-resident chondrocytes. Based on increasing metabolic activity and volume of chondrocytes, growth plate cartilage can be divided into resting, proliferative and hypertrophic zone, the latter representing the place of bone formation. Adult articular cartilage in the joint is subdivided into superficial, transitional and deep zone, with increasing proteoglycan content and decreasing cell density (Michelacci et al., 2023).

### 1.2 Cartilage diseases

Alterations in this intricate structure of the cartilage matrix are at the root of most orthopedic diseases. During development, disruptions in growth plate cartilage can severely impair skeletal growth and cause cartilage and bone deformities (Krishnan and Grodzinsky, 2018). In adult articular cartilage, altered matrix organization can cause tissue damage and impair proper function. Due to a lack of vasculature and low cellularity, articular cartilage has a limited reparative capacity, predisposing it to degenerative diseases such as different forms of arthritis (Goldring et al., 2017). Osteoarthritis (OA) is the most common form of arthritis and is characterized by pathological changes and progressive degradation of the cartilage matrix. In early stages, loss of proteoglycans and a phenotypic shift of chondrocytes alters the composition of the cartilage matrix. During disease progression, catabolic enzymes degrade the collagen network, impairing cartilage integrity. Thereby, degradation products and ECM fragments can be released, triggering inflammation and pain. This amplifies cartilage degradation and eventually results in permanent structural and functional damage (Nasiri et al., 2023).

### 1.3 Chondrocytes and cartilage matrix

As the sole resident cell type in cartilage, chondrocytes are responsible for the production and turnover of the ECM. While constituting only a small fraction of the total tissue volume, chondrocytes produce a variety of macromolecules that maintain the cartilage homeostasis (Goldring et al., 2017). Operating under hypoxic conditions, chondrocytes secrete structural ECM molecules such as collagens, proteoglycans and other glycoproteins (Coyle et al., 2009), thereby determining the structural composition of the ECM. In addition, chondrocytes produce membrane receptors such as integrins and syndecans (Behonick and Werb, 2003; Dreier et al., 2011). These provide contact points and enable communication between cells and surrounding matrix. Thereby, the ECM can affect chondrocyte function and behavior (Behonick and Werb, 2003; Gao et al., 2014).

### 1.4 Matrix production in the endoplasmic reticulum

Maintenance of the cartilage ECM creates a high demand of protein synthesis. This is reflected by the prominent endoplasmic reticulum (ER) observed in chondrocytes, particularly in the middle and deep zones of articular cartilage (Horwitz and Dorfman, 1968; Brighton et al., 1984). The ER is the largest organelle of the cell and plays a crucial role in maintaining cellular homeostasis. The ER lumen is delimited by a continuous lipid bilayer that forms a network of interconnected tubules and sheets. This membrane complex extends from the nuclear envelope to the cell periphery, spanning a large area of the cytoplasm. Depending on the cell type, the ER makes around 15% of the total cell volume (Garfield and Cardell, 1987). Morphologically, the ER can be divided into two subfractions with distinct functions. The rough ER is comprised of sheet-like membranes with ribosomes and vesicles bound to the surface. These membrane-bound ribosomes are rare or absent in the smooth ER fraction. The smooth ER has a tubular structure and is mainly responsible for the synthesis and metabolism of lipids. It also serves as a storage site for cellular calcium. In contrast, the rough ER is the main site of protein synthesis, quality control and folding of membrane and secretory proteins. It is distributed across the whole cell but is dense near the nucleus and the Golgi apparatus (Schwarz and Blower, 2016). ER structure and size are not static. Instead, it can adapt to changing conditions, e.g., during chondrocyte differentiation and ECM synthesis. Ultrastructural electron microscopy studies on embryonic and mature cartilage demonstrated an enlargement of the rough ER, a dilation of the cisternae and a condensation of the lumen in response to chondrogenesis, differentiation and other conditions of increased protein demand (Godman and Porter, 1960; Horwitz and Dorfman, 1968).

**FIGURE 1:**
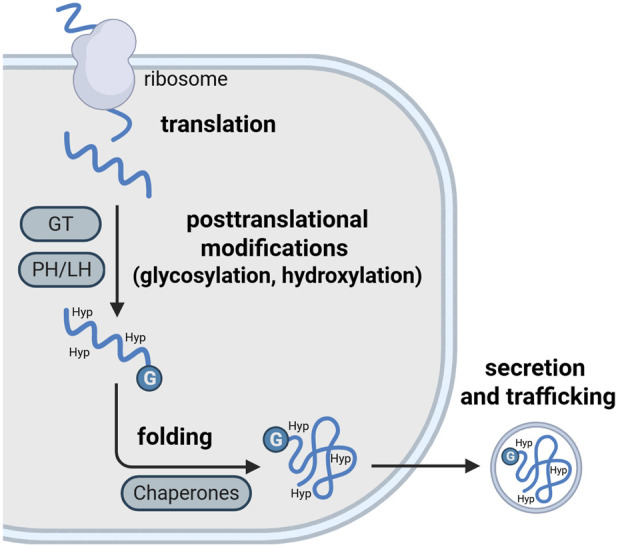
Key steps of the ECM protein processing machinery in the rough ER. Nascent proteins are translated into the rough ER by ribosomes. In the ER lumen, these proteins undergo a rang of posttranslational modifications. These include glycosylation by glycosyltransferases (GT) and hydroxylation by lysyl- and prolylhydroxylases (LH, PH) and enables the folding of newly synthesized proteins. Protein folding is facilitated by various ER-resident chaperones. Folded proteins are finally secreted and trafficked to their target sites. Image created with BioRender.com.

During protein synthesis, ribosomes are recruited to the ER and proteins are translated into the ER lumen. Inside the ER, nascent ECM proteins undergo various post-translational modifications, including glycosylation and folding. These reactions are catalyzed by a wide range of ER-resident chaperones and folding enzymes (Figure 1; Table 1). Proper folding is essential for proteins to reach their destined target site and fulfil their appropriate function (Horwitz and Dorfman, 1968; Schwarz and Blower, 2016). Therefore, disrupted protein processing in the ER can compromise the folding capacity and cause the misfolding of proteins. Misfolded proteins are retained in the ER and accumulate in the lumen. This buildup of misfolded proteins disrupts ER homeostasis and induces a state of ER stress, that compromises ER function. In response to ER stress, the ER activates various signaling pathways that are collectively referred to as the unfolded protein response (UPR). In this process, misfolded protein can be removed by autophagy and the ER-associated degradation (ERAD) complex that recognizes non-native proteins and subsequently targeting them for proteasomal degradation. If these efforts to restore ER homeostasis fail, apoptotic pathways are initiated to eliminate affected cells (Rellmann and Dreier, 2018; Sim et al., 2022).

**TABLE 1:** Molecular chaperones of the rough ER with functional significance in skeletal diseases.

ER chaperone	Function (possible substrates, examples)	Role in skeletal diseases	Literature
ERp57 (PDIA3)	Glycoprotein-specific protein disulfide isomerase (Integrin chains, Collagen α1 (VI), Laminin chains, Agrin, ADAM10, ADAM17)	Loss of ERp57: ER stress, impaired skeletal growth and early-onset OA development in mice	Jessop et al. (2009a) Wang et al. (2010) Linz et al. (2015) Rellmann et al. (2021)
ERp72 (PDIA4)	Protein disulfide isomerase, protein folding, thiol-disulfide interchange reactions (Matrilin-3, Aggrecan, Collagen type IX, COMP)	MED Pseudoachondroplasia Loss of ERp72: early-onset OA	Cotterill et al. (2005) Hecht et al. (2001) Yang et al. (2025)
ERp46 (PDIA15)	Protein disulfide isomerase (Integrin chains, Collagen α1 (VI), Laminin chains)	RA	Jessop et al. (2009b) Wang et al., 2013, 2016 Lu et al. (2020) Chang et al. (2009)
Calnexin (CNX)	Ca^2+^ binding, enables substrate recognition for ERp57, quality control of glycoproteins	Degeneration of cartilage in RA and OA Loss of CNX: ER stress, chondrocyte apoptosis	Jessop et al. (2009a) Hammond et al. (1994) Tran et al. (2025)
Calreticulin (CRT)	Ca^2+^ binding, mediates ERp57 binding to SERCA, enables substrate recognition for ERp57, quality control of glycoproteins	Reduced osteoclast activity Pro-inflammatory effects in RA	Fischer et al. (2017) Jessop et al. (2009a) Corbett et al. (1999) Li and Camacho (2004) Hammond et al. (1994) Ding et al. (2014)
BiP (GRP78)	Folds and stabilizes cartilage matrix proteins, regulator of unfolded protein response	ER stress marker in pseudoachondroplasia	Munro and Pelham (1986) Hecht et al. (2001)
HSP47	Collagen folding (Col I and II)	Kashin-Beck disease osteogenesis imperfecta osteophyte formation in OA loss of HSP47: chondrodysplasia with severe limb deformities and impaired bone mineralization	Zhang et al. (2021) Nagata et al. (1986) Masago et al. (2012) Christiansen et al. (2010) Otsuka et al. (2025)
HSP90 (GRP94)	Integrin folding ligand for TLR2 activation of NFκB signaling and cholesterol synthesis	Inflammation in OA and RA osteoclastogenesis in RA osteoporosis	De Seny et al. (2020) Huang et al. (2009) Cheng et al. (2023)
HSP72	Suppression of NFκB signaling	Reduction of RA development and progression	Luo et al. (2011)

Compromised protein folding and excess ER stress in chondrocytes has been implicated in skeletal dysplasia and various forms of arthritis. Due to the high complexity of the enzymatic landscape in the ER, many studies focus on single enzymes and their role in these pathologies. This review aims to provide an overview of the most important ER-resident proteins with their diverse functions and summarize their involvement in skeletal development, cartilage homeostasis and degeneration.

## 2 ER-resident proteins

### 2.1 Molecular chaperones

While small proteins may fold autonomously, folding of larger membrane proteins and secreted proteins (e.g., ECM proteins) is facilitated by molecular chaperones and folding enzymes in the lumen of the ER (Figure 1). By definition, folding chaperones aid the folding of non- or misfolded proteins into their native state without being part of the final protein (Halperin et al., 2014). They are among the most abundant proteins in the cell, accounting for 15%–25% of the total soluble cellular protein content (Anken and Braakman, 2005). There are multiple families of ER chaperones with both distinct and overlapping functions. The main chaperones include protein disulfide isomerases, lectins and heath shock proteins.

#### 2.1.1 Protein disulfide isomerases

**FIGURE 2:**
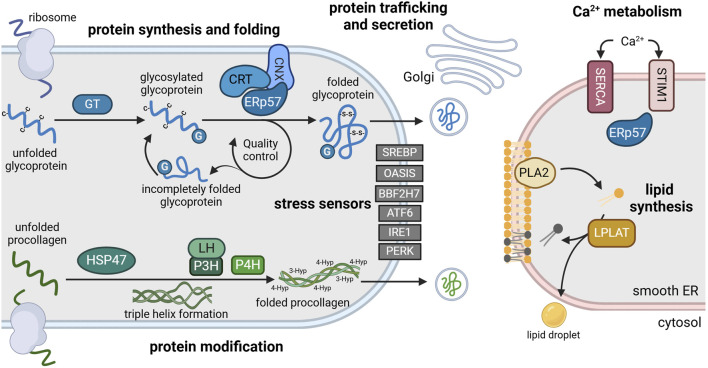
Main functions of the ER and its resident enzymes in chondrocytes. The rough ER (left) is the site of protein synthesis and processing. Here, nascent glycoproteins are translated into the lumen where they are glycosylated by glycosyltransferases (GT) (blue). Glycosylated glycoproteins (G) are recognized by a chaperone complex composed of ERp57 and calnexin (CNX) or calreticulin (CRT). This complex enables disulfide bridge formation (-S-S-) between cysteine residues (-C) that is essential for proper protein folding. Folded proteins are transported to the Golgi apparatus for secretion, while incompletely folded proteins re-enter the folding cycle. Newly synthesized procollagen is modulated by heat shock protein 47 (HSP47), lysylhydroxylases (LH) and prolyl-hydroxylases (P3H, P4H) to enable triple helix formation, folding and secretion (green). Various transcription factors including sterol element binding protein (SREBP), old astrocyte specifically induced substance (OASIS), BBF2 human homolog on chromosome 7 (BBF2H7), activating transcription factor 6 (ATF6), inositol-requiring enzyme 1 (IRE1) and pancreatic ER kinase (PERK) function as stress sensors, anchored in the ER membrane in an inactive state during homeostasis (grey). The smooth ER (right) is the main site of lipid synthesis (yellow) and Ca^2+^ storage (red). Phospholipase 2 (PLA2) and lysophosphosphatidylcholine acyltransferase (LPLAT) synthesize and remodel phospholipids that are incorporated into membranes or lipid droplets. Ca^2+^ influx is regulated by Ca^2+^ transporters such as sarco-endoplasmic reticulum calcium ATPase (SERCA) and stromal interaction molecule 1 (STIM1) via binding of ERp57. For references refer to the main text. Image created with BioRender.com.

Protein disulfide isomerases (PDI) are a large family of 21 luminal oxidoreductases with versatile functions (Ellgaard and Ruddock, 2005). PDIs catalyze the formation (oxidation), cleavage (reduction) and rearrangement (isomerization) of disulfide bonds between cysteine residues. Thereby, they aid proper folding and refolding of newly synthesized proteins and prevent the accumulation of misfolded proteins in the ER. PDI family members can undergo conformational changes depending on their redox state (Okumura et al., 2015). In order to maintain their chaperone function, PDIs are continuously re-oxidized by ER-resident oxidoreductases and peroxidases, creating reactive oxygen species in the process (Liu et al., 2024). PDI family members include ERp72 (Mazzarella et al., 1990), ERp46 and the glycoprotein-specific ERp57 (Jessop et al., 2007), which are abundantly expressed in the cisternae of the rough ER of chondrocytes (Hecht et al., 2001). ERp57 folds heavily glycosylated ECM proteins such as collagens integrins or matrix metalloproteases (Jessop et al., 2009b), which form essential components, receptors or modulators of the cartilage matrix (Figure 2; Table 1).

##### 2.1.1.1 PDIs in skeletal development

ERp57, also referred to as PDIA3, is crucial for skeletal development. While homozygous deletion of ERp57 causes embryonic lethality, heterozygous ERp57 deficiency manifested in growth plate abnormalities and decreased metaphyseal bone volume (Wang et al., 2010). Cartilage-specific knockout of ERp57 caused ER stress, defective remodeling of the ECM and chondrocyte apoptosis in the growth plate, manifesting in impaired long bone growth in young mice (Linz et al., 2015; Table 1). *In vitro*, ERp57 knockout impaired Vitamin D_3_-dependent signaling by blocking Vitamin D_3_-induced protein kinase C (PKC) activation (Wang et al., 2010), which plays an important role in bone development and regulates the secretion of matrix-degrading enzymes into the cartilage ECM (Boyan and Schwartz, 2009). In line with this, loss of ERp57 also significantly reduced bone formation *in vitro* (Chen et al., 2010).

PDIs are also involved in the processing of mutant proteins, that have been associated with different forms of skeletal dysplasia. For instance, ERp72 reportedly binds mutant forms of the collagen-binding protein matrilin-3 (Cotterill et al., 2005), resulting in its retention in the ER. Subsequent accumulation of these mutants causes epiphyseal dysplasia which often progresses to early-onset OA (Table 1). In pseudoachondroplasia, ERp72 is also involved in retaining mutant versions of cartilage oligomeric matrix (COMP), aggrecan and collagen type IX in the ER of growth plate chondrocytes (Hecht et al., 2001). This accumulation of proteins and folding enzymes causes an enlargement of the cisternae in the rough ER. In line with this, transcriptional profiling studies described an upregulation of ERp57, ERp72 and other folding proteins in hypertrophic growth plate cartilage of two mouse models carrying collagen type X misfolding mutations (Cameron et al., 2011). These studies demonstrate a vital role for PDIs in the maintenance and function of various structural components of the cartilage matrix in the growth plate.

##### 2.1.1.2 PDIs in cartilage degeneration

During postnatal growth, ERp57 is particularly important in periods with extensive matrix deposition (e.g., growth spurts) (Linz et al., 2015). In knee joints of aged mice, ERp57 knockout and subsequent ER stress induced OA-like changes, including chondrocyte apoptosis, cartilage degeneration and osteophyte formation (Rellmann et al., 2021; Table 1). Interestingly, the female sex hormone estradiol has a protective function against the effects induced by ERp57 loss. Specifically, it alleviated ER stress and subsequent apoptosis, reducing OA development and cartilage degeneration in female compared to male ERp57 knockout mice (Dreier et al., 2022).

Knockout of ERp72 also promoted OA progression *in vivo*. Mice lacking ERp72 displayed exacerbated cartilage degeneration and synovial inflammation compared to wildtype animals (Yang et al., 2025), suggesting a similar role to ERp57 in maintaining cartilage homeostasis and joint integrity.

##### 2.1.1.3 PDIs in inflammatory arthritis

PDIs are also involved in joint inflammation during various arthropathies. Elevated levels of ERp46 were detected in serum and synovial fluid of patients with RA, OA and calcium pyrophosphate deposition disease and correlated with the degree of histological synovitis (Chang et al., 2009; de Seny et al., 2020; Figure 2; Table 1). *In vitro*, ERp46 promoted the proliferation and migration of RA synovial fibroblasts and induced secretion of the inflammatory cytokines TNF-α and IL-1β (Wang et al., 2013) and the pro-angiogenic factors VEGF and IL-6 (Lu et al., 2020). Consistent with these findings, ERp46 overexpression promoted the development of collagen-induced arthritis, causing bone erosion, pannus formation and systemic inflammation (Wang et al., 2013). ERp46 also activated catabolic pathways, inducing the expression of matrix metalloproteinases that degrade the cartilage matrix (Wang et al., 2016; Figure 2). Conversely, microRNA and siRNA mediated inactivation of ERp46 reduced cytokine production, proliferation and migration of synovial fibroblasts *in vitro* (Wang et al., 2013; Wang et al., 2016). These findings highlight the complex and multifactorial involvement of PDIs in the pathogenesis of degenerative and inflammatory joint diseases.

##### 2.1.1.4 PDIs in Ca^2+^ metabolism

As an intracellular calcium storage, the ER contains a high concentration of Ca^2+^ ions (0.4–1 mM) (Corbett and Michalak, 2000; Clapham, 2007). Most of this luminal calcium is bound to ER chaperones (Macer and Koch, 1988). PDIs can also affect Ca^2+^ homeostasis by regulating designated transporters and interacting with ER-resident chaperones and Ca^2+^ sensors. In the presence of high ER Ca^2+^ levels, ERp57 also binds to the ER-resident Ca^2+^-sensor stromal interaction molecule (STIM1) which regulates store-operated Ca^2+^-influx (Prins et al., 2011; Figure 2). When ER Ca^2+^ levels are depleted, STIM1 is released and translocated to the ER membrane to induce an influx of Ca^2+^ in order to refill ER stores. Additionally, ERp57 has been shown to interact with the Ca^2+^-transporter sarco-ER calcium ATPase (SERCA) (Li and Camacho, 2004). At high ER Ca^2+^ concentrations, ERp57 binds and oxidizes SERCA, thereby inhibiting its pump activity. Consequently, Ca^2+^ depletion leads to a dissociation of ERp57, leaving SERCA in its reduced active state (Li and Camacho, 2004).

##### 2.1.1.5 PDI in skeletal mineralization

Besides its function as a signaling molecule, Ca^2+^ is a crucial component of mineralized tissues. It associates with inorganic phosphate to form hydroxyapatite, the main component of bone and calcified cartilage (Fuerst et al., 2009). Phosphate is also stored in the ER (Bublitz and Steavenson, 1988) and can be modulated by PDI. A study by Couasnay identified the phosphate transporter PiT1 as a binding partner for PDI. PiT1 is a well-established marker of mineralization (Bernabei et al., 2024) and has been shown to co-localize with PDI in the ER of growth plate chondrocytes (Couasnay et al., 2019; Table 2). Here, induction of ER stress increased both PiT1 and PDI expression *in vitro*. Conversely, deletion of PiT1 impaired PDI activity. Thereby, loss of PiT1 further promoted ER retention of aggrecan and reduced proteoglycan secretion in growth plate cartilage (Couasnay et al., 2019).

**TABLE 2:** Other rough ER-resident proteins with clinical implication in skeletal diseases.

ER protein	Biological role	Associated skeletal diseases	Literature
PiT1	Supports reductase activity of ERp46	Loss of PiT1: ER-stress and chondrocyte death during endochondral ossification	Couasnay et al. (2019)
Prolyl-4-hydroxylase (P4H)	4-hydroxylation of proline residues in collagens (Gly-X-Y motif)	Osteogenesis imperfecta Loss of PH4: chondrodysplasia	Annunen et al. (1998) Berg and Prockop (1973) Rauch et al. (2015) Syx et al. (2021)
Prolyl 3-hydroxylase (P3H)	3-hydroxylation of proline residues in collagens	Osteogenesis imperfecta	Morello et al. (2006) Syx et al. (2021)
Lysyl hydroxylase 1 (LH1)	Hydroxylation of lysine residues in collagens	Loss of LH1: no collagen fibrillogenesis and crosslinking, reduced bone mass Ehlers Danlos syndrome Morquio syndrome A with early-onset OA	Heard et al. (2016) Yeowell and Walker (2000) Syx et al. (2021) Bank et al. (2009)
Glucosyltransferases (GT) Xylosyltransferase 1 (*XYLT1*) β1,4-galactosyltransferase-7 (*B4GALT7*) N-acetylglucosaminyltransferase (*EXTL3*) chondroitin sulfate synthase 1 (*CHSY1*) N-Acetylgalactosaminyltransferase 3 (GALNT3) O-GlcNAc transferases (OGT)	ER quality control system (Tagging of incompletely folded proteins) Addition of GAGs to proteoglycan core proteins	Loss of GTs: dwarfism and skeletal abnormalities with chondrocyte hypertrophy and disrupted matrix organization in growth plate cartilage Ehlers Danlos syndrome chondrodysplasia loss of GALNT3: increased bone mass, hyperphosphataemia and extra-skeletal calcification elevated GALNT-driven glycosylation of lectins: OA and RA accumulation of O-GlcNAc-modified proteins: hypertrophy, osteophyte formation, secretion of pro-inflammatory cytokines and matrix-degrading metalloproteinases	Ellgaard and Helenius (2003) Schreml et al. (2014) Taieb et al. (2023) Guo et al. (2017) Wilson et al. (2012) Esapa et al. (2012) Tran et al. (2025) Tardio et al. (2014) Andrés-Bergós et al. (2012) Kang et al. (2025)
Solute carrier family 35 (SLC35)	Nucleotide sugar transporters	Chondrodysplasia with impaired glycan-branching and GAG synthesis Loss of SLC35: OA	Hiraoka et al. (2007) Kang et al. (2025)
Activating transcription factor 6 (ATF6)	Regulator of unfolded protein response (UPR) together with inositol-requiring enzyme-1 (IRE1) and protein kinase R-like endoplasmic reticulum kinase (PERK)	Loss of ATF6: impaired chondrogenesis and chondrocyte hypertrophy reduced mineralization and endochondral bone growth via interaction with Runx2	Xiong et al. (2015) Guo et al. (2016) Hughes et al. (2017) Rellmann et al. (2021)
OASIS	Transcription factor, promoting collagen type I transcription in osteoblasts	Loss of OASIS: impaired bone formation	Murakami et al., 2009, 2011
BBF2H7	Transcription factor important for proteins involved in vesicular trafficking of proteins from ER to Golgi	Loss of BBF2H7: severe chondrodysplasia resulting in early postnatal death	Saito et al. (2009) Asada et al. (2011)
ATF2	Osteoblast differentiation by increasing RUNX2 expression and promoting matrix mineralization	Loss of ATF2: impaired bone growth due to reduced proliferation and differentiation of growth plate chondrocytes	Ding et al. (2023) Beier et al. (2000)
Sterol regulatory element-binding proteins (SREBPs) SREBP-1	Transcription factors of the basic helix-loop-helix leucine zipper (bHLH-LZ) family that regulate genes of cholesterol and lipid metabolism induce PERK signaling regulates the transcription of frizzled-related protein 2 (Sfrp2)	Early-onset OA osteoclastogenesis and bone loss increased inflammation via transcription of pro-inflammatory mediators, NfκB activation and macrophage polarization	Yokoyama et al. (1993) Hu et al. (2020) Kim et al. (2015b) Kostopoulou et al. (2012) Cheng et al. (2023) Xu et al. (2024)

The co-presence of calcium and phosphate enables their precipitation as amorphous calcium phosphate complexes which provide the basis for mineralization. A recent imaging study located these mineral precursors in the ER of osteoblast-like cells and tracked them to the mitochondria where they accumulated to electron-dense mineral granules (Tang et al., 2020). Here, they could serve as nucleation centers and promote mineral growth. While mineralization represents a physiological process during bone formation, pathological mineralization of cartilage is a common phenomenon in aging and degenerative joint disorders such as OA. By affecting both Ca^2+^ and phosphate homeostasis, PDIs may thereby play a key role in pathophysiological mineralization, which has become a topic of interest in recent years (Zhang et al., 2024a). In this context, a recent study by Lu and colleagues demonstrated impaired mineralization, reduced collagen content and decreased bone density in a mouse model with osteoblast-specific PDI knockout (Lu et al., 2025). In fact, PDIs have been implicated in several mineralization disorders such as osteoporosis or osteogenesis imperfecta. For instance, missense mutations in the PDI-encoding gene *P4HB* lead to bone fragility and cause a form of osteogenesis imperfecta, which is characterized by skull and facial deformities (Rauch et al., 2015; Figure 4). PDIs are also highly expressed during osteoclastogenesis and have been implicated in the pathogenesis of postmenopausal osteoporosis (Wang et al., 2024; Yuan et al., 2024). In a recent study, Yuan and colleagues could suppress osteoclast differentiation and promote osteogenesis by inhibiting ERp57 activity. Thereby, they could reduce bone loss in a mouse model of postmenopausal osteoporosis (Yuan et al., 2024). This effect was mediated by a reduced expression of STIM1 and other Ca^2+^ trafficking enzymes which inhibited Ca^2+^ oscillations in osteoclast progenitor cells. A previous work by the same group showed similar beneficial effects of PDI inhibition on *in vivo* osteogenesis by reducing intracellular oxidative stress (Wang et al., 2024). These findings suggest that PDIs may affect mineralization via multiple pathways.

##### 2.1.1.6 Extracellular functions of PDI

Originally identified as ER-resident proteins, PDI have also been detected extracellularly (Chen et al., 2020; Xu et al., 2021). In chondrocyte cultures, the presence of extracellular ERp57 has been detected in the medium (Linz et al., 2015). The mechanism of secretion is still debated and the functions of extracellular PDI are not fully understood yet (Xu et al., 2021). Although the extracellular fraction of PDI is rather small, it may play a significant role in various pathophysiological processes including thrombosis, cancer and inflammation (Xu et al., 2021). However, the role in cartilage has yet to be identified.

#### 2.1.2 Lectin chaperones

The lectin family includes ubiquitously expressed ER chaperones that bind specific glycan residues to facilitate the folding and maturation of glycoproteins (Pearse and Hebert, 2010). Calnexin (CNX) and calreticulin (CRT) are the most extensively studied members of this family. They share a high Ca^2+^ binding affinity and are involved in cartilage homeostasis and disease. CNX is anchored to the ER membrane (David et al., 1993), while CRT resides in the ER lumen (Ostwald et al., 1974), binding approximately half of the Ca^2+^ content in the ER lumen (Somlyo et al., 1985). CRT and CNX bind to the PDI ERp57 to form an efficient and superior folding complex (Jessop et al., 2009a; Figure 2). When ER Ca^2+^ levels are low, CRT forms a complex with ERp57 to enable substrate recognition and binding and accelerate folding function (Zapun et al., 1998; Corbett et al., 1999). When ER Ca^2+^ stores are full, Ca^2+^ induces a conformational change in CRT. This results in a dissociation of the CRT-ERp57 complex and an increased concentration of free CRT in the ER lumen. Thereby, CRT serves as a Ca^2+^ buffer and regulates chaperone interactions within the ER (Corbett et al., 1999). In addition, CRT regulates luminal Ca^2+^ levels by mediating ERp57’s interaction with SERCA pumps (Li and Camacho, 2004; Table 1).

In association with ERp57, CRT and CNX are essential components of the CNX/CRT cycle, an ER-internal quality control system for glycoproteins (Hammond et al., 1994). They recognize and bind glucose residues on unfolded and partially folded proteins that were added by glucosyltransferases (GT) (Figure 2; Table 1). Glucosidases remove these glucose residues on correctly folded glycoproteins, terminating their interaction with CNX/CRT and enabling exit from the ER (Zapun et al., 1998; Ellgaard and Helenius, 2003). Incompletely folded proteins are “glucose-tagged” by GT again for renewed folding attempts by CNX/CRT and ERp57. Via repeated de- and re-glycosylation, nascent proteins can undergo multiple binding cycles to CRT and CNX until reaching their final conformation (Hammond et al., 1994; Sousa and Parodi, 1995).

##### 2.1.2.1 Lectins in chondrogenesis and bone formation

Developmentally, CRT has been shown to regulate the switch between chondrogenic and osteogenic fate in embryonic stem cells. CRT promotes differentiation towards the osteoblast lineage via inhibition of Glycogen synthase kinase-3 beta (GSK3β), while its absence favors chondrogenesis (Pilquil et al., 2020). Application of recombinant CRT inhibited osteoclastogenesis *in vitro* by reducing Ca^2+^ oscillations and blocking key osteoclast differentiation pathways. *In vivo*, CRT reduced osteoclast activity and osteolysis in lipopolysaccharide-induced bone inflammation (Fischer et al., 2017). Overexpression of CRT in murine chondrocyte progenitor cells reduced proteoglycan deposition and aggrecan expression, while CRT knockdown did not affect these parameters (Bomer et al., 2016).

##### 2.1.2.2 Lectins in cartilage degeneration and aging

Compressive mechanical stress reportedly increases CRT expression in rat mandibular cartilage, particularly in intermediate and deep zones. This was accompanied by increased PDI expression, ER stress and chondrocyte apoptosis (Li et al., 2013). Vice versa, CRT was downregulated in mice lacking the deiodinase iodothyronine type-2 (D2) gene (*DIO2*), a specific risk gene for OA. This was associated with reduced cartilage damage and delayed development of mechanically-induced OA (Bomer et al., 2016).

**FIGURE 3:**
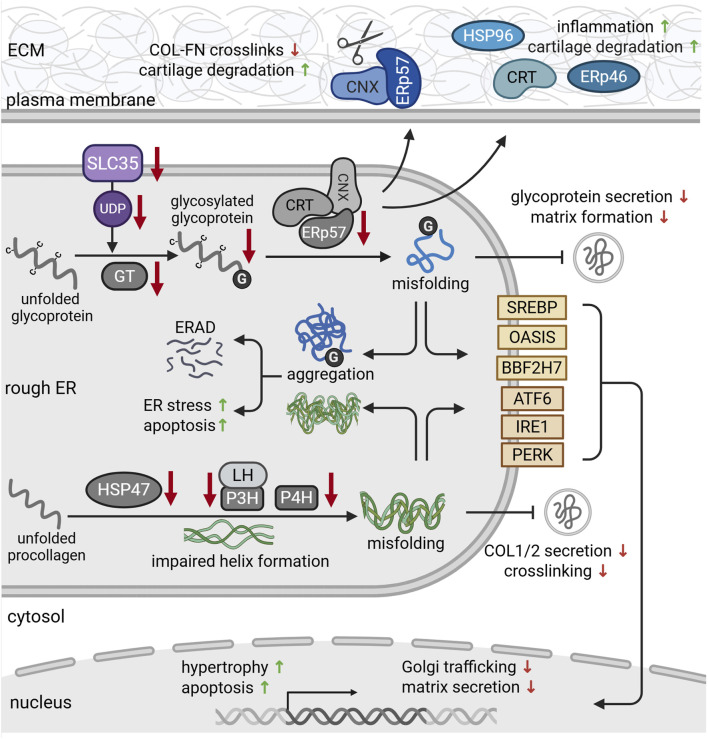
Protein dysfunction in the rough ER during chondrodysplasia and cartilage degeneration. Solute carrier family (SLC35) transporters provide uridine diphosphate (UDP)-sugars that serve as glycosylation substrates (purple). During osteoarthritis (OA), chondrocytes express reduced levels of SLC35. In addition, missense mutations in glycosyltransferases reduce glycoprotein glycosylation and glycosaminoglycan chain elongation. Reduced expression of chaperones such as ERp57, calnexin (CNX) and calreticulin (CRT) impairs the folding capacity of the ER, leading to protein misfolding. Misfolded proteins aggregate in the ER and are targeted for proteosomal degradation via ER-associated degradation (ERAD) pathways. ER retention of misfolded glycoproteins further reduces their secretion, causing ER stress and subsequent apoptosis. This eventually impairs cartilage matrix formation. During osteoarthritis (OA), CNX-ERp57 complexes (blue) can be transported to the cell surface, where they cleave disulfide bonds between collagens (COL) and fibronectin (FN), promoting cartilage degeneration. Secretion of chaperones such as calreticulin (CRT), ERp57 and heat shock protein 96 (HSP96) (blue) also triggers inflammation and cartilage degradation. Deficiencies of HSP4, prolyl- (P3H, P4H) and lysylhydroxylases impair the formation and stability of procollagen triple helices (green). This delays collagen secretion and crosslinking in the matrix and causes ER stress. Misfolded and aggregated proteins are recognized by the stress sensors in the ER membrane (yellow). Upon activation, they regulate gene expression in order to alleviate ER stress and mitigate misfolded protein load. For references refer to the main text. Image created with BioRender.com.

CNX has also been implicated in the degeneration of cartilage during arthritis (Tran et al., 2025; Table 1). Using synovial fibroblasts from OA and RA patients, the authors demonstrated increased glycosylation of CNX, leading to a translocation of the ERp57-CNX complex to the cell surface. Here, ERp57 may cleave disulfide bonds that crosslink collagen and fibronectin in the matrix (Figure 3). This disulfide bond cleavage degrades the cartilage matrix, causing arthritis-like symptoms *in vivo*. Consequently, administration of CNX-blocking antibodies could prevent these matrix-degrading effects and preserve joint integrity *in vivo* (Tran et al., 2025). On the other hand, Tan and colleagues showed increased apoptosis and ER stress marker expression in response to CNX knockdown *in vitro*. These effects resembled their findings in aged articular cartilage of monkeys that showed reduced levels of CNX and other chaperones. Overall, the efficacy of the ER folding machinery has been shown to decline with age (Kaushik and Cuervo, 2015). Thus, compromised expression and function of CNX and other chaperones during aging may promote ER stress and chondrocyte apoptosis, likely contributing to OA pathogenesis (Rellmann et al., 2021).

##### 2.1.2.3 Lectins in inflammatory arthritis

In contrast to their decline in aging and OA, CNX and CRT show an opposite trend in inflammatory arthritis. For instance, elevated levels of autoantibodies against CNX and the heat shock protein family chaperone HSP70 have been detected in the serum of RA patients (Weber et al., 2010). This upregulation of CNX was identified as an early event, occurring within the first 3 months after disease onset, and was stable over at least 12 months. As a chronic immune disease, RA manifests in inflammation of the synovial lining of the joint, angiogenesis and destruction of articular cartilage and bone (Ding et al., 2014). Similar to CNX, CRT has also been implicated in RA pathogenesis. CRT levels were upregulated in plasma and synovial fluid of RA patients (Tarr et al., 2010), correlating with disease severity (Ni et al., 2013; Figure 3; Table 1). Ding and colleagues also located high CRT expression in the synovium, particularly in regions of inflammation. They ascribed this proinflammatory effect of CRT to increased nitric oxide production and subsequent angiogenesis (Ding et al., 2014). In complex with ERp57, CRT is also involved in the folding, assembly and antigen loading of MHC molecules (Garbi et al., 2007; Blees et al., 2017). MHC variants are associated with an increased risk for severe RA and CRT has been identified as a ligand for shared epitope alleles within these variants (Ling et al., 2010). These findings indicate an important role for both CNX and CRT in RA pathogenesis and suggest their inhibition as a promising target to counteract ECM destruction in the treatment of RA.

#### 2.1.3 Heat shock proteins

Heat shock proteins (HSP) represent another major family of ER chaperones. They exert various functions, including translocation, folding and degradation of secretory and membrane proteins and are abundantly expressed in chondrocytes (Vanmuylder et al., 1997; Hecht et al., 2001). BIP or GRP78 belongs to the HSP70 protein family and is among the most extensively studied members (Munro and Pelham, 1986).

##### 2.1.3.1 HSPs in chondrogenesis and collagen synthesis

During chondrogenesis, BIP is particularly important for processing cartilage matrix proteins, e.g., COMP and colocalizes with aggregated mutant COMP that is retained in the ER during pseudoachondroplasia (Hecht et al., 2001; Table 1). HSP70 proteins have further been shown to interact with Sox9 (Marshall and Harley, 2001), a master transcription factor for chondrocyte lineage differentiation (Bi et al., 1999).

In addition to HSP70 proteins, HSP47 plays a key role in cartilage development. While most chaperones bind a broad range of substrates, HSP47 specifically aids the folding of collagen (Nagata et al., 1986). Collagen synthesis starts with the translation of procollagen molecules in the chondrocyte ER. Here, HSP47 binds to newly synthesized procollagen, stabilizing the characteristic triple-helix structure to prevent aggregation (Figure 2; Table 1). Upon transition of procollagen chains into the Golgi, HSP47 detaches and gets recycled back to the ER (Satoh et al., 1996; Ito and Nagata, 2017). Consequently, HSP47 expression correlated with collagen expression (Clarke et al., 1993). As collagens form the main structural component of the cartilage matrix (Eyre, 1991), HSP47 is indispensable for the integrity of cartilage. Its deficiency leads the aggregation of misfolded and unfolded procollagen within the ER and has been associated with various skeletal disorders (Figure 3; Table 1). For instance, cartilage-specific knockdown of HSP47 severely disrupted chondrogenesis and endochondral ossification. Mice of this genotype exhibited severe limb deformities and impaired bone mineralization, eventually resulting in perinatal lethality (Masago et al., 2012). Loss of HSP47 further reduced extracellular collagen type II and VI content and caused fibrillar misalignment in cartilage (Masago et al., 2012). Phenotypically, chondrocyte-specific knockout of HSP47 closely resembles chondrodysplasia mouse models carrying mutations in *COL2A1* (Li et al., 1995). HSP47 has further been implicated in the pathogenesis of Kashin-Beck disease, a degenerative joint disorder that mostly affects children and manifests in arthritic pain, joint deformities and growth retardation (Zhang et al., 2021). A recent study by the same group identified a downregulation of HSP47 as a driver of chondrocyte apoptosis and cartilage damage in hypertrophic chondrocyte progenitor cells and T-2 toxin treated rats (Zhang et al., 2021; Zhang et al., 2025). Specifically, toxin-induced cartilage damage and nutritional Selenium-deficiency significantly reduced cartilage expression of HSP47, thereby disrupting collagen type II synthesis and stability and promoting matrix degradation.

Besides collagen type II, HSP47 is also crucially involved in the maturation of other types of procollagens such as type I. Consistently, missense mutations in the HSP47 encoding gene *SERPINH1* cause a recessive form of osteogenesis imperfecta. In these patients, mutated HSP47 is degraded by the proteasome. While this loss of HSP47 did not affect the overall production and posttranslational modification of type I procollagen, the secretion was delayed, leading to an accumulation of procollagen type I in the ER and Golgi (Figure 3). In addition, loss of HSP47 impaired the stability of the characteristic triple helix structure, rendering procollagen molecules more susceptible to degradation by proteases (Christiansen et al., 2010). *In vitro*, HSP47 knockout resulted in aberrant collagen type I fibrillation and network formation, accompanied by reduced secretion and evident accumulation of in the ER. These effects could be reversed by transient HSP47 expression (Ishida et al., 2006). Conversely, HSP47 overexpression has recently been implicated in osteophyte formation in OA knee joints (Otsuka et al., 2025). Here, HSP47 expression was increased in TGF-β and BMP2-stimulated human osteophytic cell spheroids, while inhibition of HSP47 reduced osteophyte formation.

During procollagen maturation, HSP47 cooperates with the peptidylpropyl isomerase FKBP65, another chaperone for procollagen type I (Ito and Nagata, 2017). Still, FKBP65 cannot compensate for HSP47 deficiency. In fact, *SERPINH1* mutations not only disrupted HSP47 expression but also had similar effects on FKBP65, impairing their interaction and the processing of type I procollagen (Duran et al., 2015).

These findings highlight the importance for HSP47 in the maturation, secretion and fibrillation of various types of collagens (Figure 2). Thereby, HSP47 is a crucial chaperone for the development and integrity of the collagenous cartilage matrix.

##### 2.1.3.2 HSPs in ER stress and cartilage degeneration

HSP70 proteins are key player in ER stress and often used as a marker for this. They bind to ER stress sensors, inactivating them under basal conditions. In the presence of misfolded proteins, HSP70 proteins dissociate, activating ER stress sensors and initiating the UPR (Preissler and Ron, 2019; González-Quiroz et al., 2020). This includes an inhibition of mRNA translation, upregulated chaperone expression and increased protein degradation to deal with ER stress and mitigate the misfolded protein load (González-Quiroz et al., 2020). Naturally, HSP70 proteins are upregulated during ER stress, exerting chondroprotective effects. Transfection of chondrocytes with the HSP70 protein BIP increased proliferation, matrix production and reparative capacity (Sato et al., 2012). BIP is also upregulated in cartilage biopsies from OA patients, accompanied by increased collagen production (Nugent et al., 2009). In OA cartilage, HSP70 expression was localized in the deeper layers and correlated with disease severity (Takahashi et al., 1997). Here, HSP70 overexpression increased chondrocyte viability and protected against OA (Grossin et al., 2006). Consistent with these findings, increased ambient BIP expression was associated with delayed OA development and chondrocyte apoptosis *in vivo*, possibly due to more efficient coping with ER stress (Kung et al., 2019). A recent gene set variation analysis revealed an upregulated gene expression of several other HSPs such as HSPA5, HSPA6 and others in OA patients (Cai et al., 2025).

##### 2.1.3.3 HSPs in inflammatory arthritis

HSP90, also referred to as GRP94 or Endoplasmin, is a major chaperon for Toll-like receptors (TLRs) and integrins. By facilitating TLR and integrin folding, HSP90 optimizes B-cell function (Liu and Li, 2008). It is highly expressed in synovial tissue of RA and OA patients and its expression correlates with the degree of synovitis. HSP90 also functions as an endogenous ligand for TLR2, promoting chronic inflammation in RA (Huang et al., 2009; de Seny et al., 2020; Table 1). HSP90 expression is also increased during osteoclast differentiation. Consequently, this chaperone has recently been linked to bone loss and was upregulated in bone biopsies from osteoporosis patients (Cheng et al., 2023). Specifically, HSP90 stimulated osteoclastogenesis and bone loss via activation of NfκB and cholesterol synthesis. Vice versa, inhibition of HSP90 reduced osteoclast formation and alleviated bone loss *in vitro* and *in vivo* (Cheng et al., 2023), suggesting HSP90 as a promising target in the treatment of osteoporosis.

In contrast to HSP90, HSP72 was downregulated in RA synovial tissue and its expression inversely correlated with histological inflammation (de Seny et al., 2020). Consistent with these findings, recombinant HSP72 reduced pro-inflammatory cytokine production in RA synovial fibroblasts *in vitro* and inhibited synovitis and arthritis development and progression *in vivo*. These anti-inflammatory effects of HSP72 could be attributed to a suppression of NfκB signaling (Luo et al., 2011; Table 1).

##### 2.1.3.4 Extracellular functions of HSPs

Similar to PDI, HSPs can be released into the extracellular space (Satoh et al., 1996) and have been detected in blood and synovial fluid (Ngarmukos et al., 2020). Here, HSP70 levels correlated with radiological OA severity and have been suggested as a biomarker for predicting the disease severity. In RA, elevated synovial fluid levels of HSP96 have been reported (Huang et al., 2009) which may serve as endogenous ligand for TLRs, activating macrophages and contributing to chronic inflammation (Figure 3). However, the functions of extracellular chaperones remain a topic of ongoing research.

### 2.2 Other ER-resident proteins

#### 2.2.1 Hydroxylases

Besides chaperone-assisted protein folding, the ER performs a range of additional modifications on newly synthesized proteins. A frequent post-translational modification is the hydroxylation of lysine and proline amino acid residues by respective lysyl- and prolylhydroxylases. These modify the side chains of proline and lysine into hydroxyproline and -lysine (Heard et al., 2016; Figure 1). This hydroxylation provides attachment sites for glycans during subsequent glycosylation and is crucial for proper collagen folding and triple helix formation. Hence, disruptions in this posttranslational collagen modification machinery impair chondrogenesis and cartilage homeostasis and are associated with various connective tissue disorders (Morello et al., 2006).

##### 2.2.1.1 Prolylhydroxylases in collagen-related diseases

Prolyl-4-hydroxylases (P4H) are abundantly expressed in chondrocytes and form a tetrameric complex with PDIs. They catalyze the 4-hydroxylation of proline residues on the Y position of the collagen-specific Gly-X-Y motif (Annunen et al., 1998; Myllyharju, 2003). This 4-hydroxylation is a frequent posttranslational modification that provides thermal stability to the fibrillar collagen triple helix structure (Berg and Prockop, 1973; Figure 2; Table 2). Homozygous deletion of P4H isoform 2 had no apparent phenotypic abnormalities, but additional haploinsufficiency of P4H isoform 1 caused severe chondrodysplasia, chondrocyte apoptosis and impairments of the biomechanical stability of growth plate cartilage *in vivo*. *P4h2−/− P4h1+/−* mice had reduced tibial bone volume, collagen content and osteoblast numbers (Aro et al., 2015; Tolonen et al., 2022), indicating the importance of P4H for skeletal development and chondrocyte survival. In line with this, P4H1 deficiency causes a connective tissue disorder that includes joint hypermobility, muscle weakness, skeletal dysplasia and myopia (Zou et al., 2017). In contrast, P4H synthesis seems to be increased during early OA, presumably due to the upregulated collagen production by hypertrophic chondrocytes in this stage of the disease (Grimmer et al., 2006). However, its specific role in cartilage degeneration remains elusive.

Hydroxylation of proline to 3-hydroxyproline is less frequent than 4-hydroxylation, particularly in collagen type I and type II which are the main types expressed in bone and cartilage (Morello et al., 2006). It usually occurs in the X position of the Gly-X-Y motif and is catalyzed by collagen prolyl 3-hydroxylases (P3H) to prevent premature procollagen aggregation in the ER (Vranka et al., 2004; Hudson and Eyre, 2013; Table 2). P3H exists in three isoforms (P3H1, P3H2, P3H3), with P3H1, also known as Leprecan, being the most abundant in cartilage (Wassenhove-McCarthy and McCarthy, 1999). Inactivating mutations in the P3H1-encoding gene *LEPRE1* (Cabral et al., 2007) have been shown to cause a severe form of osteogenesis imperfecta characterized by decreased bone density, impaired mineralization and shortened long bones. Mechanistically, loss of P3H1 reduced 3-hydroxylation, delayed helix formation and impaired fibrillogenesis of collagen type I (Figure 3). This was accompanied by increased lysyl hydroxylation due to overmodification by lysyl hydroxylases (Morello et al., 2006; Cabral et al., 2007). While P3H seems to be crucial for proper collagen I fibrillation and bone development, its role in cartilage and collagen type II maturation remains unknown.

##### 2.2.1.2 Lysylhydroxylases in collagen-related diseases

Lysyl hydroxylases (LH) catalyze the hydroxylation of lysine residues in the Y position of the Gly-X-Y motif. LH isoform 1 associates with P3H and synaptonemal complex 65 (SC65) to hydroxylate lysine at cross-linking sites in the collagen triple helical region (Heard et al., 2016; Figure 2; Table 2). Depleting this lysine hydroxylation via SC65 knockout disrupted collagen fibrillogenesis and crosslinking, causing skin fragility and reduced bone mass *in vivo* (Heard et al., 2016). LH1 mutations have further been identified as a cause for Ehlers Danlos syndrome (EDS). EDS is a connective tissue disorders of defective collagen metabolism that manifests in joint hypermobility, scoliosis and skin fragility (Hyland et al., 1992). These mutations impair the cross-linking of collagen, thereby reducing its tensile strength (Yeowell and Walker, 2000; Figure 3). In contrast, elevated LH expression in human synovium biopsies has been associated with increased collagen cross-linking and OA-related fibrosis (Remst et al., 2013). In osteogenesis imperfecta, elevated levels of LH, P3H and P4H, have been detected in patient fibroblasts. This upregulation was associated with impaired binding of HSP47 to procollagen type I and disrupted triple helix stability, presumably representing an overcompensation therefor (Syx et al., 2021). In contrast, lysyl hydroxylation levels were decreased in cartilage biopsies of Morquio syndrome A patients, which contributes to the early development of OA that is characteristic for this lysosomal storage disease (Bank et al., 2009).

#### 2.2.2 Glycosyltransferases and sugar transporters

Glycosylation is the most common posttranslational modification. It constitutes a complex multistep process of attaching sugars to asparagine (N-glycans) and serine or threonine residues (O-glycans) to form extensively branched glycan structures. These reactions are catalyzed by approximately 200 different glycosyltransferases (GTs) (Ellgaard and Helenius, 2003) and require the supply with glycosylation substrates by dedicated nucleotide sugar transporters in the ER (Kang et al., 2025). Glycosylation mostly affects secretory proteins and is important for folding and secretion (Figure 1). It also diversifies the proteome and regulates cell signaling, interaction and adhesion (Schjoldager et al., 2020). As part of the ER quality control system, GT tags incompletely folded proteins by adding a glucose molecule (Figure 2). This prevents premature exit of incompletely folded proteins from the ER and enables renewed interaction with chaperones and folding enzymes. Thereby, GT functions as a folding sensor, regulating the retention of proteins in the ER (Ellgaard and Helenius, 2003).

##### 2.2.2.1 Sugar transporters

Uridine diphosphate (UDP)-sugars serve as glycosylation substrates and are transported to the ER and Golgi by nucleotide sugar transporters of the solute carrier family (SLC35). Here, UDP-sugars are used to synthesize proteoglycan core proteins and their attached glycosaminoglycan (GAG) chains. Due to the abundant expression of proteoglycans in cartilage, chondrocytes maintain appropriate intracellular levels of UDP-sugars (Qu et al., 2007). Mutations in SLC35 transporters reduce substrate availability for ER-resident GT (Figure 3). This impairs glycan-branching and GAG synthesis, manifesting in chondrodysplasia phenotypes (Hiraoka et al., 2007). In line with this, a recent study detected reduced expression of SLC35 in OA chondrocytes which correlated with loss of proteoglycans (Kang et al., 2025; Table 2).

##### 2.2.2.2 GTs in skeletal development

Similarly, GT mutations can disrupt normal skeletal development. For instance, missense mutations in xylosyltransferase 1 (*XYLT1*) which initiates the addition of GAGs to proteoglycan core proteins, are associated with dwarfism and skeletal abnormalities (Schreml et al., 2014; Taieb et al., 2023). Mechanistically, loss of Xylt1 reduced proteoglycan glycosylation and GAG chain elongation, promoting chondrocyte hypertrophy and disrupting matrix organization in growth plate cartilage (Taieb et al., 2023; Figure 3; Table 2). Mutations in β1,4-galactosyltransferase-7 (*B4GALT7*) (Seidler et al., 2006), N-acetylglucosaminyltransferase (*EXTL3*) (Guo et al., 2017) and chondroitin sulfate synthase 1 (*CHSY1*) (Wilson et al., 2012) show similar effects on proteoglycan glycosylation and ECM structure. These mutations have been implicated in skeletal and connective tissue disorders such as EDS and chondrodysplasia which are reviewed elsewhere (Paganini et al., 2019; Dubail and Cormier-Daire, 2021). In contrast, inactivating mutations of the polypeptide N-Acetylgalactosaminyltransferase 3 (GALNT3) are associated with increased production of bone matrix. These GALNT3 mutations lead to increased bone mass, hyperphosphataemia and extraskeletal calcification mediated by reduced FGF23 glycosylation (Esapa et al., 2012). These findings demonstrate complex and heterogeneous functions of GTs in cartilage and bone development.

##### 2.2.2.3 GTs in degenerative arthritis

Gene expression analysis of human OA cartilage showed an upregulation of various GTs involved in synthesis, substitution and branching of N- and O-glycans. This was associated with distinct glycophenotypes depending on the degree of cartilage degeneration (Toegel et al., 2013). Altered N-glycation patterns detected by mass spectrometry and HPLC analysis even preceded OA-changes in human cartilage samples (Matsuhashi et al., 2008). A recent study by Tran and colleagues also found elevated O-glycosylation of lectins driven by ER-resident GALNTs in synovial membrane biopsies of OA and RA patients (Tran et al., 2025). These findings suggest a role for aberrant GT function in OA pathogenesis.

Glycosylation is not restricted to secreted proteins of the cartilage matrix. Intracellular proteins can also be glycosylated by addition of single O-linked GlcNAc monosaccharides. These are attached by O-GlcNAc transferases (OGT) and can be readily removed by specific glucosaminidases (OGA) (Chatham et al., 2021). In OA cartilage, increased O-GlcNAcylation and elevated OGT expression have been detected (Tardio et al., 2014). Similarly, O-GlcNAcylation was also upregulated during hypertrophic differentiation of chondrocytes (Andrés-Bergós et al., 2012), which represent one of the hallmark changes in OA cartilage (Table 2). In fact, accumulation of O-GlcNAc-modified proteins alone induced hypertrophy (Andrés-Bergós et al., 2012). Corroborating these findings, a recent study by Kang and colleagues detected increased O-GlcNAcylation in OA cartilage which promoted the secretion of pro-inflammatory cytokines and matrix-degrading metalloproteinases (Kang et al., 2025). Interestingly, pharmacological inhibition of OGT reduced O-GlcNAcylation, alleviated cartilage destruction, suppressed synovitis and reduced osteophyte formation in a mouse model of OA (Kang et al., 2025). Conversely, inhibition of OGA increased O-GlcNAcylation and accelerated OA progression. Thus, OGT-mediated O-GlcNAcylation may play a driving role in OA pathogenesis, potentially providing a new target for therapeutic interventions.

Downstream targets of OGT in OA cartilage include the lipid metabolism gene ACSF (Zhang et al., 2024b) and the NLRP3 activator NEK7 (He et al., 2024). Upstream regulators driving elevated OGT expression in OA cartilage mostly remain elusive. Several studies have identified inflammatory mediators such as IL1α and TNF-α to promote OGT expression in OA chondrocytes (Tardio et al., 2014) and RA synovial fibroblasts (Kim H. B. et al., 2015), respectively. OGT activity has further been linked to insulin during hypertrophic differentiation of ATDC5 cells (Andrés-Bergós et al., 2012). The regulation of OGT activity in OA chondrocytes and cartilage remains to be investigated.

#### 2.2.3 Transcription factors

Besides enzymes for protein folding and post-translational processing, the ER contains a range of transcription factors that guard its homeostasis. Disruption of the complex ER folding machinery can interfere with protein folding and cause an accumulation of misfolded proteins in the ER lumen. Attempting to restore ER homeostasis, these membrane-anchored transcription factors regulate signaling in response to ER stress. Activating transcription factor 6 (ATF6), inositol-requiring enzyme-1 (IRE1) and protein kinase R-like endoplasmic reticulum kinase (PERK) are the most well-established transcription factors. They represent the three major axes of ER stress transduction (Figure 2), activating downstream signaling pathways that are collectively referred to as the unfolded protein response (UPR). In addition to their function as ER stress transducers, they are also important regulators of skeletal development and function (Horiuchi et al., 2016). For instance, ATF6 regulates chondrogenesis and promotes chondrocyte hypertrophy, mineralization and endochondral bone growth via interaction with Runx2 (Xiong et al., 2015; Guo et al., 2016; Table 2).

The role of the ATF6, IRE1 and PERK axes of the UPR in cartilage pathophysiology has been extensively reviewed elsewhere (Hughes et al., 2017; Rellmann et al., 2021). Instead, we will focus on a few lesser-known transcription factors and their implications for cartilage homeostasis, including members of the OASIS and BBF2H7 families.

##### 2.2.3.1 BBF2H7 and OASIS in chondrogenesis

OASIS and BBF2H7 transcription factors that are structurally homologous to ATF6 (Horiuchi et al., 2016). In contrast to the ubiquitous expression of ATF6, OASIS and BBF2H7 are preferentially expressed in osteoblasts and chondrocytes, regulating their respective differentiation and exerting important functions in skeletal development (Murakami et al., 2009; Asada et al., 2011). OASIS is crucial for bone formation by promoting collagen type I transcription in osteoblasts. Thus, deletion of OASIS severely reduced bone density and osteoblast activity (Table 2). This was accompanied by an expansion of the ER and an accumulation of bone matrix proteins (Murakami et al., 2009; Murakami et al., 2011). These effects could be rescued by overexpressing OASIS in osteoblasts (Murakami et al., 2011), highlighting its requirement for proper bone formation during development. Similarly, ATF2, a family member of ATF6, also plays a crucial role in osteoblast differentiation by increasing RUNX2 expression and promoting matrix mineralization (Ding et al., 2023). ATF2 deficiency therefore reduced proliferation and differentiation of growth plate chondrocytes and impaired bone growth *in vivo* (Beier et al., 2000; Table 2).

BBF2H7 structurally resembles OASIS but is mainly expressed in chondrocytes during long bone development (Saito et al., 2009; Asada et al., 2011; Table 2). Thus, its absence causes severe chondrodysplasia with disrupted matrix production, organization and composition, resulting in early postnatal death (Saito et al., 2009). Growth plate cartilage missing BBF2H7 had a narrowed hypertrophic zone with reduced ECM proteins. In the proliferating zone, chondrocytes lacked the characteristic columnar organization and contained enlarged rough ER with aggregations of collagen type II and COMP. Targets of BBF2H7 include proteins that are involved in vesicular trafficking of proteins form ER to Golgi which is particularly important for chondrocytes to cover the high demand of ECM protein secretion during development (Asada et al., 2011; Figure 3). Additionally, BBF2H7 is able to suppress chondrocyte apoptosis in growth plate cartilage by activating other ER-resident transcription factors such as activating transcription factor 5 (ATF5) (Izumi et al., 2012). These findings indicate a bifunctional role for BBF2H7 in chondrogenesis via stimulation of ECM production and suppression of ER stress-induced apoptosis.

##### 2.2.3.2 Sterol regulatory element-binding proteins in chondrogenesis, degenerative and inflammatory arthritis

**FIGURE 4:**
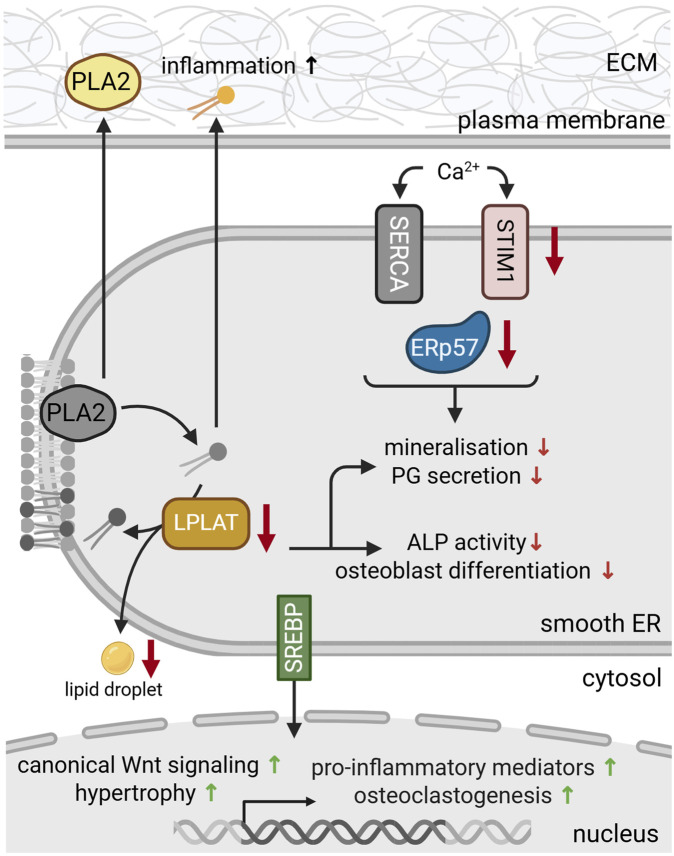
Protein dysfunction in the smooth ER during skeletal dysplasia and bone pathologies. During differentiation, reduced levels of LPLAT inhibit proteoglycan (PG) secretion and osteoclastogenesis. LPLAT deficiency (yellow) further reduced alkaline phosphatase (ALP) activity and bone mineralization and impaired lipid droplet formation. Deficiencies in Ca^2+^ transporters and sensors such as stromal interaction molecule 1 (STIM1) (pink) and ERp57 (blue), respectively, disrupt the luminal Ca^2+^ metabolism, aggravating dysfunctional mineralization and matrix secretion. ER stress and deficient luminal lipid levels activate membrane bound sterol regulatory element-binding proteins (SREBP) (green). Upon translocation to the nucleus, SREBPs regulate gene expression to stimulate lipid synthesis, while also promoting canonical Wnt signaling, hypertrophic differentiation, osteoclastogenesis and the transcription of pro-inflammatory mediators. Secreted phospholipase 2 (PLA2) and phospholipids (yellow) also promote the production of pro-inflammatory mediators, contributing to inflammation and OA progression. For references refer to the main text. Image created with BioRender.com.

Sterol regulatory element-binding proteins (SREBPs) are transcription factors of the basic helix-loop-helix leucine zipper (bHLH-LZ) family that regulate genes involved in cholesterol and lipid metabolism (Yokoyama et al., 1993). They exist in three isoforms and are synthesized in inactive precursor forms. Inactive precursor SREBPs remain bound to the ER membrane in the presence of lipids. When lipid levels are depleted, SREBPs are proteolytically cleaved and released from the ER to enter the nucleus (Yokoyama et al., 1993). Here, they activate the transcription of genes for lipid synthesis and uptake (Yang et al., 2000). SREBPs can also induce PERK signaling and have been linked to ER stress and autophagy via PERK response pathways (Hu et al., 2020; Figure 3). During chondrogenesis, SREBP-1 and SREBP-2 were upregulated in ATDC5 cells (Allendorf, 2009). In this context, SREBP-1 has been shown to regulate the transcription of frizzled-related protein 2 (Sfrp2) (Kim M.-J. et al., 2015) that supports canonical Wnt signaling, an important signaling pathway in cartilage homeostasis and bone formation (de Castro et al., 2021; Figure 4).

SREBPs have also been investigated in the context of OA, as this is often accompanied by lipid metabolism alterations (Xu et al., 2024; Table 2). Genetic association studies of OA patients identified single nucleotide polymorphisms in the SREBP gene that elevated SREBP protein levels and promoted OA development (Kostopoulou et al., 2012). In line with this, SRBEP expression was upregulated in articular cartilage and chondrocytes of OA patients compared to healthy controls. This upregulation was accompanied by reduced SOX9 and collagen type II levels as well as upregulated levels of TGF-β, MMP13 and collagen type X, marking increased chondrocyte hypertrophy (Kostopoulou et al., 2012; Tao et al., 2015; Figure 4). In intervertebral disc degeneration, active SREBP was essential for cholesterol-induced ER stress and promoted ECM degradation and cell death via pyroptosis (Yan et al., 2021). SREBP had similar catabolic effects on bone, promoting osteoclastogenesis and bone loss upon activation by HSP90 (Cheng et al., 2023).

Further, SREBPs can promote inflammation via the transcription of pro-inflammatory mediators, NfκB activation and macrophage polarization (Xu et al., 2024; Figure 4). In gout, SREBPs contribute to monosodium urate crystal-induced inflammation via YAP signaling. SREBP overexpression promoted hyperuricemia and endothelial inflammation, while pharmacological inhibition of SREBP alleviated gout-induced inflammation (Zhao et al., 2021). These findings suggest SREBP as a promising therapeutic target in degenerative and inflammatory joint diseases.

#### 2.2.4 Lipogenic enzymes

While glycoproteins constitute the large majority of the cartilage matrix, lipids make up only 1% of the total weight (Stockwell, 1967). Nonetheless, they are essential for various cellular and mechanical processes in the joint, including chondrocyte differentiation, mineralization and joint lubrication (Steinmeyer, 2025). Above all, lipids such as phospholipids and sphingolipids are essential components of cell membranes. Phospholipids contain a glycerol backbone, whereas sphingolipids are built on a sphingosine base. Both are synthesized in the smooth ER by different enzymes and multiple intermediates (Tidhar and Futerman, 2013). Substrates for phospholipid synthesis include phosphatidylcholine and phosphatidylethanolamine. Phospholipase A2 (PLA2) and lysophosphatidylcholine acyltransferase (LPLAT) are key enzymes in the synthesis of phospholipids, responsible for their de- and re-acylation (Figure 2). Precursors of sphingolipids include serine and ceramide, relying on serine palmitoyltransferases (SPTLC) and ceramide synthases (Steinmeyer, 2025). While there is progress in unraveling the complex lipid metabolism in cartilage, there is limited data on ER-specific changes in lipid synthesis.

##### 2.2.4.1 Lipogenic enzymes in chondrogenesis and osteogenesis

As a key enzyme in phospholipid synthesis, LPLATs have been shown to regulate chondrocyte differentiation and mineralization. Specifically, LPLAT4 expression and activity increased during chondrogenic differentiation of ATDC5 cells, particularly in the stage of hypertrophy and mineralization (Tabe et al., 2017). LPLAT isoform 2 also mediates the formation of lipid droplets during osteoblast differentiation (Figure 2). In line with this, LPLAT2 knockdown inhibited the expression of chondrogenic markers, decreased proteoglycan content and reduced lipid droplet number (Tabe et al., 2024). Loss of LPLAT4 further inhibited alkaline phosphatase (ALP) activity, which is a commonly used marker for mineralization. Recently, the same group has detected increased expression of LPLAT2 and associated BMP/Smad activation during osteoblast differentiation (Tabe et al., 2024). Consistently, LPLAT2 knockdown inhibited osteoblast differentiation and reduced ALP activity (Figure 4). Thus, LPLATs modulate differentiation by activating osteo- and chondrogenic signaling pathways.

##### 2.2.4.2 Lipogenic enzymes in degenerative and inflammatory arthritis

Alterations in the lipid metabolism are also commonly reported in arthritic joints and have been established as a key player in OA pathogenesis (Tsezou et al., 2010; Yang et al., 2021). For instance, increased expression of PLA2 was detected in articular cartilage and synovium of OA patients (Pruzanki et al., 1991; Leistad et al., 2011). PLA2 hydrolyses membrane phospholipids to release free fatty acids and lysophospholipids that can be re-acylated by LPLATs (Kita et al., 2019; Figure 2). As PLA2 is a key enzyme in phospholipid remodeling, it is not surprising that elevated phospholipid levels have been detected in OA synovial fluid (Eichner et al., 2025). This effect was already detected in early stages of the disease, preceding radiological changes. Therefore, lipidomic alterations may be a promising indicator for early OA development (Eichner et al., 2025). In addition to its involvement in lipid synthesis in the ER, PLA2 itself can also be secreted, having been detected in synovial fluid of OA and RA patients (Pruzanki et al., 1991). Extracellular PLA2 has pro-inflammatory activities, promoting the production of inflammatory mediators such as prostaglandins (Leistad et al., 2011; Figure 4). In line with this, PLA2 expression positively correlated with histological inflammation (Jin et al., 2024) and nanoparticle-mediated inhibition of PLA2 was able to reduce inflammation and slow down OA progression (Wei et al., 2021).

Similarly, SPTLC, a key enzyme in sphingolipid synthesis, is highly expressed in OA cartilage, particularly in the deep zone (Mori et al., 2014; Lü et al., 2023). Accordingly, elevated levels of sphingolipid precursors have detected in OA synovial fluid (Kosinska et al., 2013). These were associated with chondrocyte apoptosis and matrix degradation (Sabatini et al., 2000). However, SPTLC subunit 2 (SPTLC2) reportedly serves chondroprotective functions. Lentiviral overexpression of SPTLC2 in articular cartilage and enhanced chondrocyte viability, decreased apoptosis and increased cell numbers (Lü et al., 2023). SPTLC2 overexpression also increased the expression of ECM proteins, while inhibiting the expression of catabolic matrix metalloproteinases. Thereby, SPTLC2 enhanced matrix integrity and protected against cartilage degradation. Silencing of SPTLC2 caused opposite effects, promoting chondrocyte apoptosis and matrix degeneration (Lü et al., 2023). Although there is limited data available, the beforementioned studies suggest that lipid-synthesizing enzymes may crucially contribute to cartilage pathophysiology, affecting chondrogenesis, mineralization and inflammation.

## 3 Summary and conclusion

Hyaline cartilage is a tissue with unique biomechanical properties and versatile function during skeletal development and function. It contains a dense ECM that mainly consist of large glycoproteins and is produced by chondrocytes. To meet the high protein demand, chondrocytes have a distinct rough ER that serves as the major site of protein synthesis, folding and post-translational processing. Here, the ER harbors a vast collection of enzymes that catalyze versatile post-translational modifications to secure proper folding and maturation of newly synthesized ECM proteins. These enzymes guide skeletal development and are essential for the formation and function of the cartilage matrix.

Loss or malfunction of ER-resident proteins in skeletal cells can affect a multitude of cellular processes, including gene expression, protein secretion and ECM function. This can have far-reaching consequences for cartilage, bone and the entire skeletal system. Altered gene expression and cell differentiation in growth plate cartilage can disrupt chondrogenesis and bone formation. In addition, imbalances in Ca^2+^ metabolism can affect cell signaling and impair mineralization during skeletal development, manifesting in chondrodysplasia and bone deformities.

Reduced chaperone function during aging impairs the secretion and crosslinking of ECM proteins (e.g., collagens and proteoglycans). Disrupted ECM production compromises cartilage organization and stability, rendering the tissue susceptible to degradation by proteases (e.g., MMPs). Aberrant processing and secretion of proteins also causes ER stress. Excess ER stress can induce apoptosis, further contributing to cartilage loss during aging and OA.

It is clear that ER-resident proteins are crucial for viability and function of chondrocytes and other cells of the skeletal system. However, due to the high complexity of the ER protein processing machinery, the specific roles of ER-resident enzymes in skeletal homeostasis are incompletely understood. Their contribution to skeletal diseases also remains elusive and remain a promising topic for future research.
